# Gliotoxin Isolated from Marine Fungus *Aspergillus* sp. Induces Apoptosis of Human Cervical Cancer and Chondrosarcoma Cells

**DOI:** 10.3390/md12010069

**Published:** 2013-12-24

**Authors:** Van-Tinh Nguyen, Jung Suck Lee, Zhong-Ji Qian, Yong-Xin Li, Kil-Nam Kim, Soo-Jin Heo, You-Jin Jeon, Won Sun Park, Il-Whan Choi, Jae-Young Je, Won-Kyo Jung

**Affiliations:** 1 Department of Biomedical Engineering, and Centre for Marine-Integrated Biomedical Technology (BK21 Plus) Pukyong National University, Busan 608-737, Korea; E-Mail: nvtinh@pknu.ac.kr; 2 Industry-Academic Cooperation Foundation, Jeju National University, Jeju 690-756, Korea; E-Mail: jungsucklee@hanmail.net; 3 College of Food Science and Technology, Guangdong Ocean University, Zhanjiang 524088, China; E-Mail: qianzhongji@hanmail.net; 4 Marine Bioprocess Research Center, Pukyong National University, Busan 608-737, Korea; E-Mail: lyxycg@hotmail.com; 5 Marine Bio Research Team, Korea Basic Science Institute (KBSI), Jeju 690-140, Korea; E-Mail: roadman5969@hanmail.net; 6 Global Bioresources Research Center, Korea Institute of Ocean Science & Technology, Ansan 426-744, Korea; E-Mail: sjheo@kiost.ac.kr; 7 Department of Marine Life Sciences, Jeju National University, Jeju 690-756, Korea; E-Mail: youjinj@jejunu.ac.kr; 8 Department of Physiology, Kangwon National University School of Medicine, Chuncheon 200-701, Korea; E-Mail: parkws@kangwon.ac.kr; 9 Department of Microbiology, College of Medicine, Inje University, Busan 608-737, Korea; E-Mail: cihima@inje.ac.kr; 10 Department of Marine Bio-Food Sciences, Chonnam National University, Yeosu 550-749, Korea

**Keywords:** apoptosis, *Aspergillus* sp., gliotoxin, human cervical cancer cells, human chondrosarcoma cells

## Abstract

Gliotoxin, a secondary metabolite produced by marine fungus *Aspergillus* sp., possesses various biological activities including anticancer activity. However, the mechanism underlying gliotoxin-induced cytotoxicity on human cervical cancer (Hela) and human chondrosarcoma (SW1353) cells remains unclear. In this study, we focused on the effect of gliotoxin induction on apoptosis, the activating expressions of caspase family enzymes in the cells. Apoptotic cell levels were measured through DAPI and Annexin V/Propidium Iodide (PI) double staining analysis. The apoptotic protein expression of Bcl-2 and caspase family was detected by Western blot in Hela and SW1353 cells. Our results showed that gliotoxin treatment inhibited cell proliferation and induced significant morphological changes. Gliotoxin induced apoptosis was further confirmed by DNA fragmentation, chromatin condensation and disrupted mitochondrial membrane potential. Gliotoxin-induced activation of caspase-3, caspase-8 and caspase-9, down-regulation of Bcl-2, up-regulation of Bax and cytochromec (cyt c) release showed evidence for the gliotoxin activity on apoptosis. These findings suggest that gliotoxin isolated from marine fungus *Aspergillus* sp. induced apoptosis in Hela and SW1353 cells via the mitochondrial pathway followed by downstream events leading to apoptotic mode of cell death.

## 1. Introduction

Apoptosis, a major form of cell death, is characterized by several unique features, including cell shrinkage, nuclear collapse, membrane blebbing, and internucleosomal DNA cleavage (DNA fragmentation) [1,2]. Programmed cell death plays critical roles in a wide variety of physiologic processes during fetal development and in adult tissues [3]. Defects in apoptosis facilitate tumor progression, by rendering cancer cells resistant to death mechanisms relevant to metastasis, growth factor deprivation and chemotherapy [4]. The evidences were gradually accumulated that many cancer chemotherapeutic agents killed the cancer cell by inducing apoptosis. Mainly two apoptotic pathways are known as the intrinsic (death receptor-mediated) and the extrinsic (mitochondrial-mediated) pathway [1]. In the intrinsic pathway, mitochondria play a key role in mediating apoptosis; opening of the permeability transition pore and a subsequent drop in mitochondrial membrane potential (ΔΨm) have been suggested as the main mechanisms [2]. Mitochondrial damage is associated with the induction of caspases and reactive oxygen species production. Loss of ΔΨm leads to the release of cytochrome c (cyt c) from mitochondria, leading to the activation of caspase-9 and further activating the downstream effector caspase-3 [5].

Caspase activation is a widely accepted pathway of cell death. Caspases also cleave a variety of substrates involved in activities that lead to dismantling of the cell such as disruption of organelle function, cytoskeletal, and nuclear disassembly, resulting in the typical hallmark features of apoptotic cell death [6,7]. Caspase-3 activation is an important step in apoptosis execution [8]. Pro- and anti-apoptotic proteins are members of Bcl-2 family, which are found to be up-regulated (Bax) and down-regulated (Bcl-2) in a number of apoptosis. Translocation of Bax to mitochondria results in the release of cyt c into cytosol. The tumor suppressor p53 induces apoptosis via several mechanisms [9]. The p53 is able to activate cell cycle progression, DNA repair and apoptosis [10,11].

To date, the cervical carcinoma is the second most common cancer in women, and is one of the major causes of death among women in the world [5,12]. Chondrosarcoma is a malignant primary bone tumor and the third most common primary malignancy of bone after myeloma and osteosarcoma [13,14]. Thus, we chose human cervical cancer cells (Hela) and human chondrosarcoma cells (SW1353) for the study.

**Figure 1:**
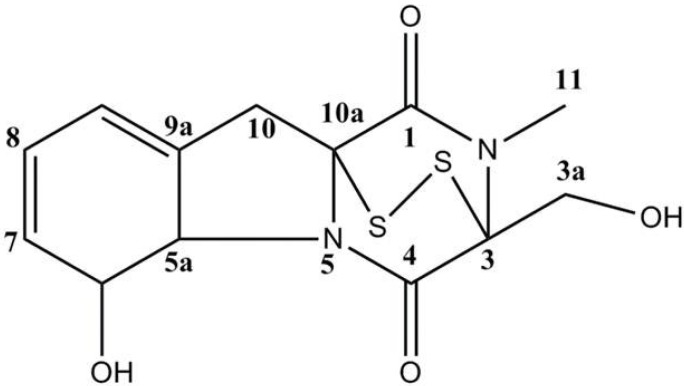
Chemical structure of gliotoxin from *Aspergillus* sp.

Marine-derived fungi have proved to be a promising source of bioactive metabolites and a growing number of marine fungi have been reported to produce bioactive secondary metabolites [15,16]. *Aspergillus* species are filamentous saprophytic fungi that can be found in almost all aerobic environments. They have been found to produce a wide range of complex metabolites, some of which have important commercial application potentials. Several fungal metabolites isolated from *Aspergillus* sp. It has been shown to exert antitumor, antiinflammator, induced cytotoxicity and antibacterial activity [17]. One of them, gliotoxin, belongs to the family of epipolythiodioxopiperazines that is characterized by a disulfide bridge across a piperazine ring (Figure 1). Gliotoxin, one of the secondary metabolites produced by a number of *Aspergillus*, *Gliocladium* and *Penicillium* species, is a tricyclic alkaloid [18,19,20]. Gliotoxin is an inducer of apoptotic cell death in a number of cell types [21,22,23]. It has been found to be associated with some diseases attributed directly or indirectly to fungal infections.

The major purpose of the present study was to determine the effect of gliotoxin on Hela and SW1353 cells to evaluate its anticancer potential. In this study, we demonstrated that gliotoxin actively induced apoptosis and reduced proliferation of Hela and SW1353 cells. Our results suggest that gliotoxin induces apoptosis through mitochrondrial-dependent caspase pathway.

## 2. Results and Discussion

### 2.1. Cytotoxicity of Gliotoxin

**Figure 2:**
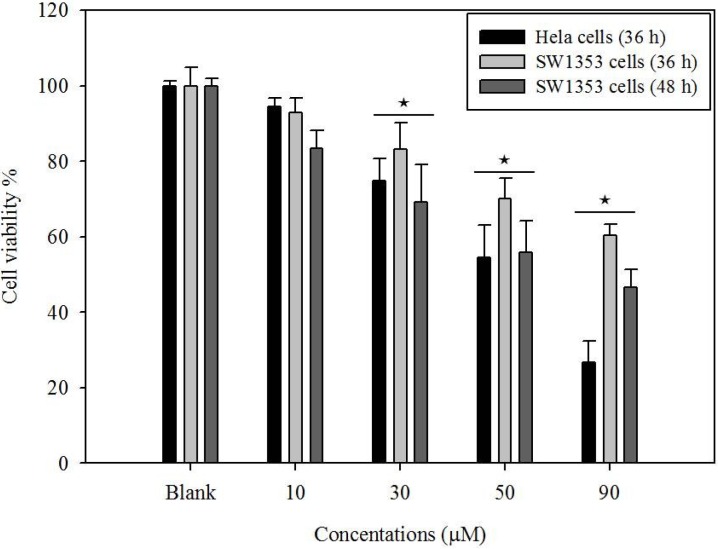
Effects of gliotoxin on the viability of Hela and SW1353 cells. Cells were treated with gliotoxin at 10, 30, 50, and 90 µM. The cell viability was determined by MTT assay. Results of independent experiments were averaged and represented as percentage cell viability. Values represent means ± SE (*n* = 3) (★ *P* < 0.05).

The 3-(4,5-dimethylthiazol-2-yl)-2,5-diphenyltetrazolium bromide (MTT) assay was performed to measure the viability inhibitory effect of gliotoxin on the Hela and SW1353 cells. It can also be used to determine cytotoxicity of potential medicinal agents and toxic materials. Those agents would stimulate the inhibition of cell viability and growth. The Hela and SW1353 cells were treated with different concentrations of gliotoxin. As shown in Figure 2, gliotoxin treatment inhibits cell growth of Hela (73%), SW1353 (39%) cells at 36 h and SW1353 cells (59%) at 48 h. Thus, SW1353 cells were incubated with gliotoxin at 48 h for each experiment. The gliotoxin showed strong antiproliferative activity in a dose-dependent manner, by presenting relative HeLa cells viabilities of 94%, 74%, 54%, and 27%, and SW1353 viabilities of 83%, 69%, 56%, and 41% at concentrations of 10, 30, 50, and 90 µM, respectively, compared to the control group. Here, cytotoxicity effect on Hela cells was higher than that on SW1353 cells’ treatment with gliotoxin at 90 µM of concentration.

### 2.2. DAPI Staining of Hela and SW1353 Cells with Gliotoxin

**Figure 3:**
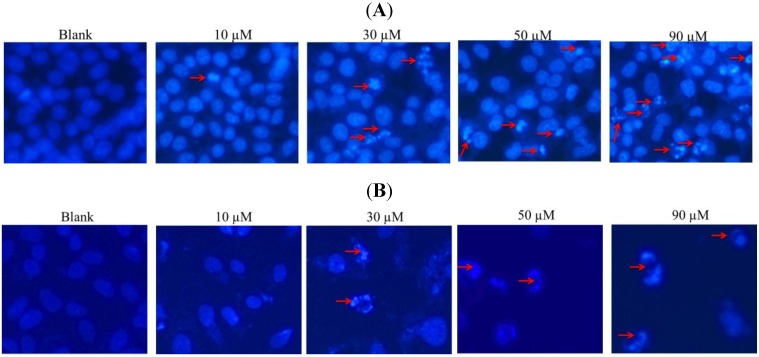
Induction of apoptosis by gliotoxin treatment in Hela (**A**) and SW1353 (**B**) cells. After being treated with 10, 30, 50, and 90 µM of gliotoxin, the cells were fixed and stained with DAPI. Stained nuclei with DAPI solution were then photographed with a fluorescent microscope using a blue filter.

DAPI staining revealed that nuclei with chromatin condensation and apoptotic bodies were formed in cells that were cultured with gliotoxin. As shown in Figure 3, the cells were stained with DAPI dye and observed under a fluorescence microscope. Viable cells (control group) with intact DNA and just slightly activated in the fluorescence microscope image were negative to DAPI. Furthermore, DAPI positive cells and their intensities were increased in a gliotoxin dose-dependent manner. This indicates that most of the cells underwent cell death primarily through apoptosis by the treatment of gliotoxin.

### 2.3. Induction of DNA Fragmentation

**Figure 4:**
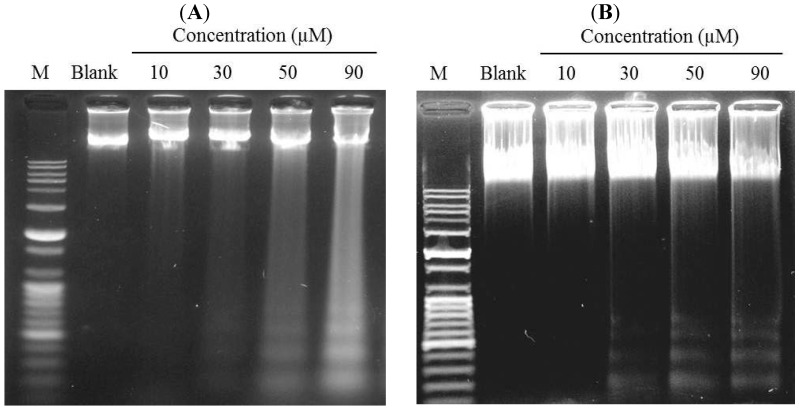
Detection of DNA fragmentation in Hela (**A**) and SW1353 (**B**) cells treated with different concentrations (10, 30, 50, and 90 µM) of gliotoxin. The genomic DNA was extracted, electrophoresed in a 1.2% agarose gel and and visualized by ethidium bromide staining under ultra-violet light. Lane M is a 100 bp plus DNA ladder.

Yang *et al.* (2006) [8] reported that measurement of the molecular weights of the fragments is consistent with internucleosomal cleavage characteristic of apoptosis. Characteristic ladder bands can be obtained by agarose gel electrophoresis of DNA extracted from apoptotic cells. In Figure 4, DNA ladder bands could be observed at and above 30 µM gliotoxin-treated group and reached a maximal level in the 90 µM gliotoxin-treated group in Hela and SW1353 cells, and vehicle treated cells did not show any DNA fragmentation in assays. These phenomena demonstrated that gliotoxin induced apoptosis in Hela and SW1353 cells in a dose-dependent manner.

### 2.4. GuL Effects on Hela and SW1353 Cells Membrane Phosphatidylserine

**Figure 5:**
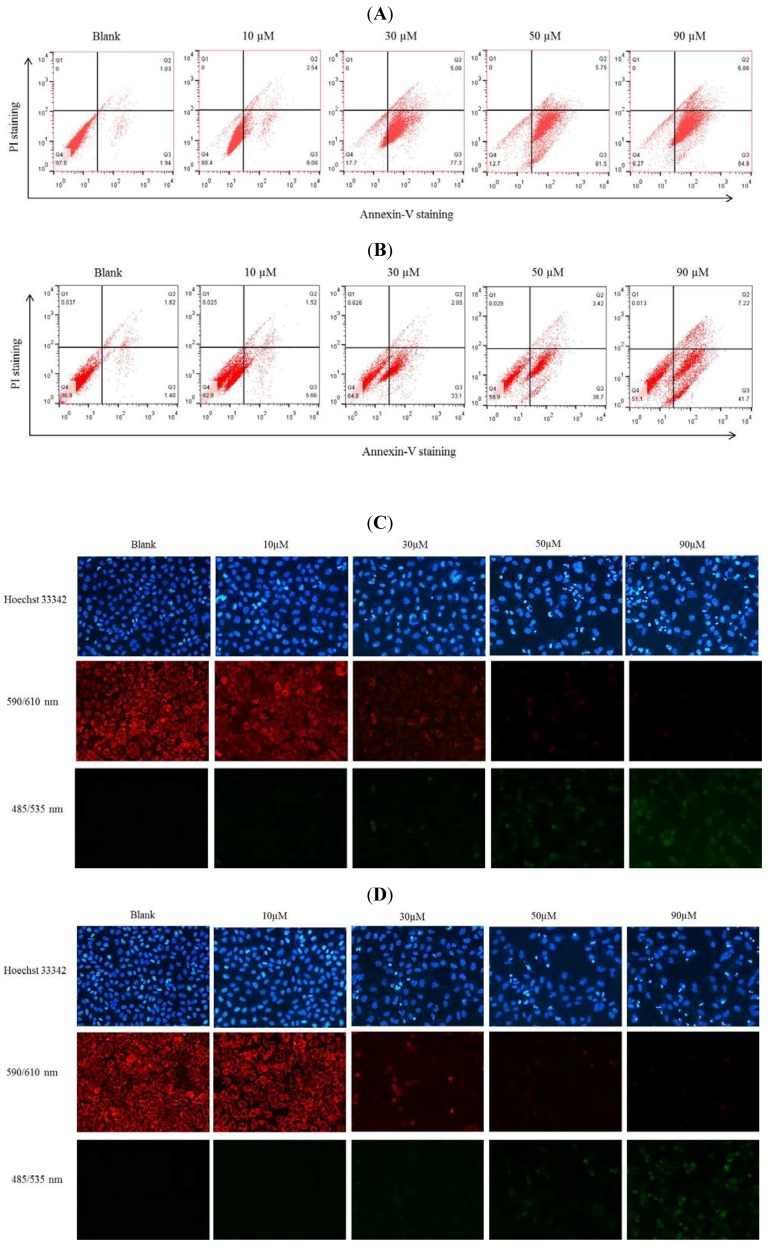
Hela (**A**) and SW1353 (**B**) cells were incubated with 10–90 μM gliotoxin or without gliotoxin *in vitro* and analyzed by Annexin V/PI staining. Frames were divided into four quadrants: Q3 represents apoptotic cells positive for Annexin V and negative for PI (Annexin V^+^ and PI^−^); Q2 represents late apoptotic and early necrotic cells (Annexin V^+^ and PI^+^); Q4 represents normal cells (Annexin V^−^ and PI^−^); and Q1 represents cells undergoing necrosis (Annexin V^−^ and PI^+^). Cell ΔΨm dissipation induced by gliotoxin. Hela (**C**) and SW1353 (**D**) cells were exposed to gliotoxin at 10–90 μM and stained by JC-1 staining and Hoechst 33342; Red fluorescence represents the mitochondrial aggregation form of JC-1 indicating intact ΔΨm. Green fluorescence represents the monomeric form of JC-1 indicating a dissipation of ΔΨm.

To determine whether the growth inhibitory effect of gliotoxin is associated with cell death, Annexin V/ Propidium Iodide (PI) double staining of Hela and SW1353 cells and flow cytometric analyses were performed. As shown in Figure 5A,B, the number of apoptotic cells increased in a dose-dependent manner after incubation with 10, 30, 50 and 90 µM gliotoxin. The upper left (Q1) quadrant of the cytograms shows the dead cells, with positive to PI and Annexin V negative (Annexin V^−^ and PI^+^). The lower left (Q4) quadrant of the cytograms shows the viable cells (Annexin V and PI negative). In the upper right (Q2) quadrants, it shows the late apoptotic and early necrotic cell (Annexin V^+^ and PI^+^) population increases. The lower right (Q3) quadrant shows apoptotic cells (Annexin V^+^ and PI^−^). Here, apoptotic cells’ effect on Hela cells was higher than that on SW1353 cell treatment with gliotoxin at 90 µM of concentration, compared to the control group. These results demonstrate that the inhibition of cell growth by gliotoxin was due to the induction of apoptosis.

### 2.5. Gliotoxin Disrupted *Δ*Ψm in Hela and SW1353 Cells

Depletion of ΔΨm is one of the early events that occur following induction of cellular apoptosis. In this work, upon incubation of Hela and SW1353 cells with gliotoxin (0–90 µM), the ΔΨm was determined. The gliotoxin-treated cells showed progressive loss of red JC-aggregate fluorescence and appearance of green monomer fluorescence in the cytoplasm at 50 and 90 µM. We found that gliotoxin treatment attenuated the ΔΨm level, and this decrease occurred in a dose-dependent manner (Figure 5C,D). These data suggest the involvement of the intrinsic pathway of apoptosis in the mechanism of cell death induction by the gliotoxin.

### 2.6. Effects of Gliotoxin on Protein and Gene Expression Levels in HeLa and SW1353 Cells

**Figure 6:**
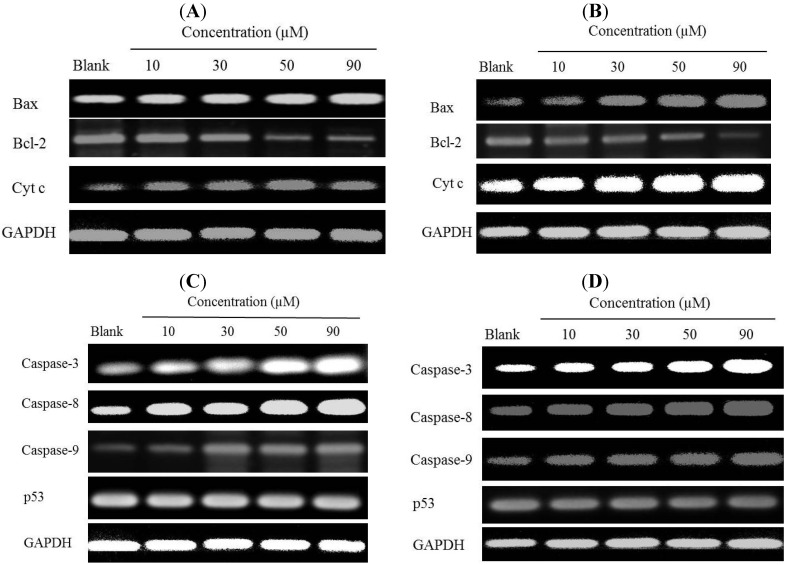
Effects of gliotoxin on the mRNA expression levels of cyt c, Bcl-2 and Bax of the HeLa (**A**) and SW1353 (**B**) cells using RT-PCR analysis. Effects of gliotoxin on the mRNA expression levels of caspase-3, caspase-8 and caspase-9 and p53 of the HeLa (**C**) and SW1353 (**D**) cells. The cells were treated with various concentrations (10, 30, 50, and 90 µM) of gliotoxin. GAPDH was used as an internal control.

To determine whether cyt c, Bax, and Bcl-2 was involved in modulating apoptosis induced by gliotoxin, we investigated the effects of gliotoxin on mRNA and protein expressions of cyt c, Bax, and Bcl-2 in HeLa and SW1353 cells. Different concentrations of gliotoxin were added to HeLa and SW1353 cells. In the RT-PCR results, gliotoxin treatment promoted the mRNA expression of cyt c, down-regulated Bcl-2 and up-regulated Bax expressions in a dose-dependent manner (Figure 6A,B). Collectively, these results suggest that gliotoxin induced apoptosis by activating expression of Bax and inhibiting Bcl-2 in both the cell lines.

To elucidate the possible mechanisms of apoptosis by activating caspase family in HeLa and SW1353 cells, we investigated the effects of gliotoxin with respect to mRNA expression by RT-PCR. As shown in Figure 6C,D, the expression levels of caspase-3, caspase-8, and caspase-9 increased at the concentrations of 10, 30, 50, and 90 µM compared to the untreated group, and continuously increased dose-dependently. However, gliotoxin did not induce the increased gene expression levels of p53.

**Figure 7:**
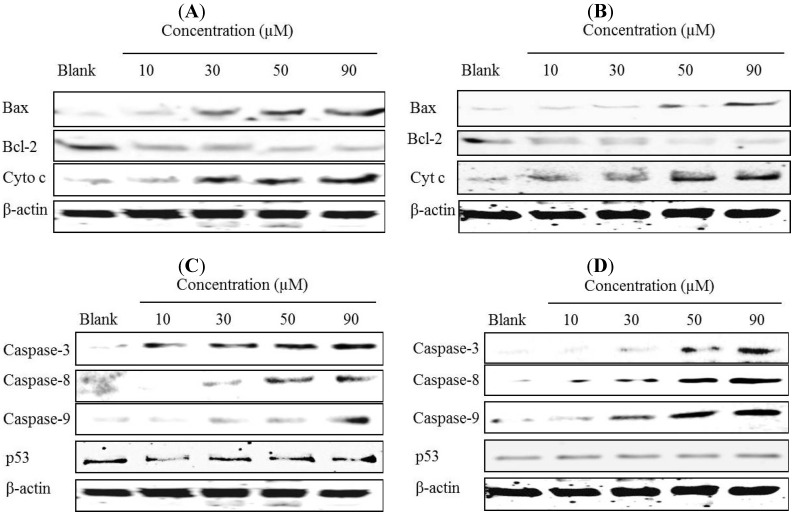
Effects of gliotoxin on apoptosis-related proteins. Western blotting analysis of cyt c, Bcl-2 and Bax of the HeLa (**A**) and SW1353 (**B**) cells treated with 10, 30, 50, and 90 µM of gliotoxin, respectively. Western blotting analysis of cleavage forms of caspase-3, caspase-8 and caspase-9, and p53 of the HeLa (**C**) and SW1353 (**D**) cells treated with 10, 30, 50, and 90 µM of gliotoxin, respectively. Band intensity was normalized to β-actin expression by densitometry.

Moreover, Western blotting analysis was used to investigate the effects of gliotoxin on cyt c, Bax, and Bcl-2 protein expression. To examine this step in the apoptotic cell death pathway initiated by gliotoxin, cyt c content in cytosol of HeLa and SW1353 cells treated with 10, 30, 50, and 90 µM of gliotoxin was measured. As shown in Figure 7A,B, the release of cyt c was initiated with only 10 and 30 µM of gliotoxin treatment. To elucidate further the possible mechanism underlying the gliotoxin-induced apoptosis, the expression of Bcl-2 and Bax in HeLa and SW1353 cells was examined after gliotoxin treatment. Exposure of HeLa and SW1353 cells to 10, 30, 50, and 90 µM of gliotoxin led to an obvious decrease of Bcl-2 protein expression, but a drastic increase of Bax protein expression in a dose-dependent manner.

Furthermore, the mechanism by which gliotoxin induces apoptosis is being actively investigated. Western blotting analysis was used to investigate the effects of gliotoxin on caspase family protein expression in Hela and SW1353 cells. Gliotoxin treatment promoted cleavage forms of caspase-3, caspase-8, and caspase-9 protein expressions dose-dependently in the HeLa and SW1353 cells (Figure 7C,D). However, it was interesting that gliotoxin almost had no effect on p53 protein expressions.

### 2.7. Discussion

The present study has shown that gliotoxin induced apoptosis via the mitochondrial pathway in HeLa and SW1353 cells. Gliotoxin could effectively induce programmed cell death, suggesting that it could be a promising anticancer. The previous study investigated the cytotoxicity induced by gliotoxin in human hepatic stellate, rat Kupffer and SH-SY5Y cells [18,21,22]. In this study, we reported for the first time an evaluation of the effects of gliotoxin on the growth of Hela and SW1353 cells *in vitro*. Similar to the previous studies, our results demonstrated that gliotoxin significantly inhibited the growth of Hela and SW1353 cells in a concentration-dependent manner by MTT assay (Figure 2). Apoptosis is a highly conserved phenomenon that plays an important role in the regulation of the cellular activities of eukaryotes and is characterized by chromatin condensation [2,23]. In our study, the toxicity in cultured Hela and SW1353 cells induced by gliotoxin was found to be due to apoptosis, since cells changed nuclear morphology by chromatin condensation using DAPI stain (Figure 3A,B). Several studies reported that apoptosis in the Hela and SW1353 cells presented typical apoptotic morphological changes with cell shrinkage, nuclei blebbing, chromatin condensation, and formation of apoptotic bodies [5,14]. Because the appearance of apoptotic bodies is regarded as the apoptotic hallmark, the results suggested that gliotoxin induced the death of Hela and SW1353 cells mainly through apoptosis. In addition, the biochemical hallmark of apoptotic cell death is the cleavage of chromosomal DNA at internucleosomal sites into fragments or multiples of about 180 bp [6]. A ladder-like pattern, a typical character of DNA cleavage between nucleosomes, was visible during incubation with gliotoxin. However, the intensity of banding was more prominent at 50 and 90 µM (Figure 4). Similarly, Qu *et al.* (2004) found that apoptosis cell death is due to cleavage of chromosomal DNA in Hela cells [24]. Moreover, in apoptosis cells, the membrane phospholipid phosphatidyserine is translocated from the inner to the outer leaflet of the plasma membrane, and is redistributed to the extracellular surface [11,22]. Surface exposure of phosphatidylserine by apoptotic cells was measured by adding Annexin V. PI has been used to identify dead or late apoptotic cells because the membranes of damaged and dead cells are permeable to PI [13]. Viable cells are negative to Annexin V and PI. However, early apoptotic cells are Annexin V positive and PI negative, and apoptotic or already dead cells are positive to both Annexin V and PI. We discovered that the media contaminant gliotoxin could increase apoptosis in Hela and SW1353 cells. It seems controversial that gliotoxin increases early apoptosis both Hela and SW1353 cells, but had more effect in Hela cells than in SW1353 cells. Note that different cell types may respond to gliotoxin differently. Here, treatment of the cells with gliotoxin for 36 h (Hela cells) and 48 h (SW1353 cells) increased the number of Annexin V^+^/PI^−^ stained cells (Figure 5A,B). Moreover, the loss of mitochondrial membrane potential, the release of pro-apoptotic factors as cytochrome c from mitochondria into the cytoplasm, and activation of the initiator caspase-9 activate the intrinsic pathway of apoptosis [9]. Our data clearly demonstrates that treatment with 10–90 µM gliotoxin could lead to a loss of ΔΨm in a dose-dependent manner in Hela and SW1353 cells (Figure 5C, D). Li *et al.* (2012) demonstrated that loss of ΔΨm induced apoptosis in SW1353 cells [25]. A similar mechanism of apoptosis induction was also found by loss of ΔΨm in Hela cells [26]. This means that gliotoxin-induced apoptosis is related to the collapse of ΔΨm, then release of cyt c from mitochondria, and the activation of apoptosis proteins such as caspases.

Apoptotic pathways can be divided into two major groups, such as extrinsic or intrinsic death [1,5]. The release of cyt c from mitochondria is tightly regulated by a variety of factors, such as Bcl-2 and Bax [4]. In addition, over-expression of Bcl-2 has been reported to protect tumor cells from apoptosis, whereas increased Bax expression promotes apoptosis via mitochondria [8]. Pardo *et al.* (2006) showed that gliotoxin induces apoptotic cell death by activating the proapoptotic Bcl-2 family member Bak, but not Bax, followed by generation of reactive oxygen species (ROS) after 4 h of treatment in mouse embryonic fibroblast [27]. A recent report also indicates that treatment with 1 μM of gliotoxin could induce apoptosis by significant involvement of Bak rather than Bax in BEAS-2B cells [28]. However, our study shows that gliotoxin induced apoptosis on Hela (after 36 h of treatment) and SW1353 cells (after 48 h of treatment) by activating expressions of Bax and inhibiting Bcl-2 in a dose-dependent manner (Figure 6 and Figure 7). We noticed a gradual increase in Bax protein levels, which may be due to some different mechanisms and the effects of gliotoxin on different concentrations and times or/and cell lines. Hence, when SW1353 cells were treated with gliotoxin for 48 h, the gradual increase of Bax was different with Hela cells for 36 h. This further leads to caspase activation and thus results in apoptosis. The mitochondrial permeability transition (MPT) results in an abrupt increase in the permeability of the inner mitochondrion membrane, and release of cyt c. Release of cyt c from the intermembrane spaces of the mitochondria into the cytosol is a key event in apoptosis [12,26]. Anselmi *et al.* (2007) and Kweon *et al.* (2003) have previously reported that gliotoxin induced apoptosis of activated human HSCs, Kupffer and macrophages cells with induction of cyt c release [21,22]. A critical cellular target of gliotoxin may be the MPT because thiols have been reported to play a functional role in the regulation of the MPT and gliotoxin through its disulfide bridge across a piperazine ring, which is known to covalently react with protein thiols [29]. In this study, we demonstrated that the mitochondrial pathways were mediated by down-regulation release of cyt c. Thus, in order confirm the results, molecular detection of apoptosis related proteins expressions including Bax, Bcl-2, and release of cyt c were carried out. Moreover, the p53 tumor suppressor protein has a critical role in regulation of the Bcl-2 family of proteins [7]. Members of the BCl-2 family can be divided into two subfamilies: the anti-apoptotic proteins include Bcl-2, Bcl-x, Bcl-XL, Bcl-XS, Bcl-w and BAG, and the pro-apoptotic proteins include Bcl-10, Bax, Bak, Bid, Bad, Bim, Bik, and Blk [2]. Several proteins can direct inhibition of the anti-apoptotic protein Bcl-2 as Bim, Puma, Bad, HrkDP5 and Noxa, whereas direct stimulation of the pro-apoptotic protein Bax as tBid and p53. In this study, gliotoxin did not change the expression of p53, and the chemical of gliotoxin-induced apoptosis via regulation of Bcl-2 family was direct stimulation or inhibition on Bcl-2 or/and Bax protein in Hela and SW1353 cells. The results accorded well with the previous data and showed that treatment with gliotoxin could up-regulate the expression of Bax, down-regulate Bcl-2 expression and release cyt c, all in a dose-dependent manner.

A variety of toxins and chemicals induce cytotoxicity via caspase. Several papers have also reported the importance of gliotoxin as induction of membrane permeability transition and caspase-3 activation [18,22,30]. Caspases are expressed in almost all cell types as inactive proenzymes. Much evidence suggests that activation of caspases triggers the apoptotic process in various cell types [24]. Activated caspase-3 has important roles in the occurrence of typical biochemical and morphological changes in apoptotic cells. The present study has provided direct evidence that caspase-3 was involved in cell death potentiated. Gliotoxin shows a number of broad toxic and inhibitory effects directed towards immune cells and the induction of apoptosis, which requires the extracellular presence of the epipolythiodioxopiperazine ring [23]. Taken together, gliotoxin-induced apoptosis is relevant to mitochondrial-mediated apoptosis via caspase-dependent pathways, elucidated by the loss of mitochondrial membrane potential, release of cyt c, and activation of Bax, caspase-3, caspase-8 and caspase-9, as well as suppression of Bcl-2. However, there was no change in p53 activity compared with the control.

## 3. Experimental Section

### 3.1. Materials

Dulbecco’s modified Eagle’s medium (DMEM), trypsin-EDTA, penicillin/streptomycin, fetal bovine serum (FBS) were obtained from Gibco BRL, Life Technologies (Grand Island, NY, USA). Hela and SW1353 cells were obtained from American Type of Culture Collection (Manassas, VA, USA). Ribonuclease A solution and proteinase K were purchased from Sigma-Aldrich (St. Louis, MO, USA). Primary and secondary antibodies used for Western blot analysis were caspase-3, caspase-8, caspase-9, Bax, Bcl-2, cyt c, p53, β-actin, goat antirabbit IgG-HRP, goat antimouse IgG_1_-HRP, and purchased from Santa Cruz Biotechnology, INC (Santa Cruz, CA, USA). Hoechst 33342 was purchased from Santa Cruz Biotechnology, INC (Santa Cruz, CA, USA). Other chemicals and reagents used were of analytical grade.

### 3.2. Extraction and Isolation of Gliotoxin

The fungal strain (stock no: YL-06) was isolated from the surface of the marine brown alga collected in the Ulsan City, Korea and identified as an *Aspergillus* sp. The fungal strain was stored in the 10% glycerol YPG (Yeast extract-peptone-glycerol) medium at −75 °C as previously described [31]. The further culture for investigation was completed on YPG medium from 10 mL to large scale (1.0 L and 10.0 L). The fungus was cultured (30.0 L) for 30 days at 29 °C in YPG medium. The culture broth and mycelium were separated, and the filtered broth was extracted with ethyl acetate to provide the broth extract (1.58 g), which was fractionated by silica gel chromatography (*n*-hexane/EtOAc) to generate six fractions. The further purification of the active fractions by ODS column chromatography (H_2_O in MeOH), followed by HPLC (YMC ODS-A, MeOH), yielded compounds gliotoxin (23.0 mg). Finally, the purified compounds were identified by ^1^H and ^13^C NMR data, which were recorded at 400 MHz, with reference to the solvent signals (Table 1). The purity of compounds was >97%, based on the peak area of all components absorbed at each specific wavelength in HPLC analysis. The compounds were dissolved in dimethylsulfoxide (DMSO) and employed in experiments in which the final concentration of DMSO in culture medium was adjusted to <0.01%. The chemical structure of gliotoxin used in the present study is shown in Figure 1.

**Table 1:** ^1^H and ^13^C NMR spectral data for gliotoxin in CDCl_3_^a^.

Position	δ_H_	δ_C_
1		166.0 (s)
3		77.2 (s)
3a	3.44 (1H, dd, *J* = 4.8), 4.28 (1H, dd, *J* = 9.9)	60.5 (t)
4		165.2 (s)
5a	4.39 (1H, dd, *J* = 6.8)	69.8 (d)
6	4.84 (1H, m )	75.6 (d)
7	5.78(1H, d, *J* = 9.9)	129.9 (d)
8	5.95 (1H, m)	123.4 (d)
9	6.00 (1H, m)	120.2 (d)
9a		130.7 (s)
10	2.96 (1H, d, *J* = 18.1), 3.73(1H, d, *J* = 18.1)	36.6 (t)
10a		73.1 (s)
11	3.20 (3H, s)	27.5 (q)

^a^ Recorded at 400 MHz for ^1^H and 100 MHz for ^13^C.

### 3.3. Cell Culture and Treatment

The cells were cultured in DMEM supplemented with 10% FBS and antibiotics. Then cells were incubated under a fully humidified atmosphere and 5% CO_2_ at 37 °C. The Hela and SW1353 cells were seeded at concentration of 1 × 10^4^ cells/well in a 96-well plate, 2 × 10^5^ cells/well in a 24-well plate and 1 × 10^6^ cells/well in a 6-well plate [8,13]. They were added 24 h after seeding and treated with different concentrations gliotoxin. After 36 h with Hela cells and 48 h with SW1353 cell incubation, the cells were harvested for each experiment.

### 3.4. Cell Viability Assay

Cell viability was determined by a colorimetric MTT assay, as previously described [11,13]. Briefly, the culture medium was changed to the experimental medium supplemented with gliotoxin at different concentrations. After incubation and treatment, cells were incubated with 50 µL of 1 mg/mL MTT reagent for 4 h. Mitochondrial succinate dehydrogenase in live cells converts MTT into visible formazan crystals during incubation. The formazan crystals were then solubilized in dimethyl sulfoxide (DMSO) and the optical density was measured at 540 nm by using a microplate reader. Relative cell viability was calculated compared to the non-treated blank group. The data were expressed as means of at least three independent experiments.

### 3.5. Nuclear Staining with DAPI

The 4,6-diamidino-2-phenylindole (DAPI) staining was carried out as previously described [1]. Briefly, cells seeded in 24-well plate were treated with different concentrations gliotoxin. After incubation, the cells were washed three times in phosphate-buffered saline (PBS) and fixed with 1 mL methanol solution at room temperature for 10 min. The fixed cells were then washed with PBS and stained with a DAPI solution (Santa Cruz, CA, USA) at room temperature in the dark. The nuclear morphology of the cells was examined by fluorescent microscopy (Carl Zeiss MicroImaging GmbH, Jena, Germany).

### 3.6. Annexin V-FITC Labeling

Apoptosis in Hela and SW1353 cells was measured by using the Annexin V/PI kit (BD Pharmingen, San Diego, CA, USA), as described previously by Wang *et al.* (2011) [11]. Briefly, Hela and SW1353 cells were exposed to different concentrations of gliotoxin in 6-well plates, were washed twice with PBS solution and then rewashed cells in binding buffer [0.01 M Hepes/NaOH (pH 7.4), 0.14 M NaCl, 2.5 mM CaCl2]. Subsequently, cells were stained with Annexin V/PI (100 µL binding buffer, 5 µL Annexin and 5 µL PI) at room temperature in the dark for 15 min. Culture dishes were thereafter rinsed twice with cold PBS and then the sample detected by flow cytometry. The different labeling patterns in the Annexin V/PI analysis where Annexin V negative and PI negative were designated as viable cells; Annexin V positive and PI negative as early apoptotic cells; Annexin V positive and PI positive as late apoptotic cells or necrotic cells; and Annexin V negative and PI positive as necrotic cells.

### 3.7. DNA Fragmentation Assay

Cells seeded in 6-well plate were harvested, washed with PBS, lysed with 120 µL RIPA buffer (50 mM Tris-HCl (pH 7.5), 150 mM NaCl, 1% Triton X-100, 1% sodium deoxycholate, 2 mM EDTA (pH 8.0), and 0.1% SDS) supplemented with proteinase K (10 mg/mL) and digested for 1 h with RNase A (10 mg/mL). After two extractions with phenol and one extraction with phenol/chloroform/isoamylacohol (25:24:1), the DNA was precipitated with 0.1 volumes of 3 M ammonium acetate and 2 volumes of absolute ethanol at −20 °C overnight, and then washed with ice-cold 70% ethanol, air-dried, and resolved in 50 µL TE buffer (10 mM Tris, 1 mM EDTA-Na_2_, pH 8.0) [32]. Ten µg of DNA was separated in a 1.5% agarose gel and visualized by ethidium bromide staining under ultra-violet light. The intensity of the bands was estimated using ZoomBrowser EX software.

### 3.8. Measurement of Mitochondrial Membrane Potential (*Δ*Ψm)

The ΔΨm was determined using the mitochondria-specific lipophilic cationic fluorescence dye 5,5′,6,6′-tetrachloro-1,1′,3,3′-tetraethylbenzimi-dazolylcarbocyanine iodide (JC-1) Detection Kit according to the manufacturer’s instructions (Abnova Corporation, Walnut, CA, USA). Hela and SW1353 cells were treated with various concentrations of gliotoxin (10, 30, 50 and 90 μM). After then, cells were treated with 100 µL JC-1 for 30 min at 37 °C in the dark. Finally, cells were washed twice with PBS and then the fluorescence intensity was examined by fluorescent microscopy (Carl Zeiss MicroImaging GmbH, Goettingen, Germany). The green monomeric form of JC-1 is excited at 485 nm and emits at 535 nm; the red aggregate form is excited at 590 nm and emits at 610 nm. The dye JC-1 can selectively enter into mitochondria. In healthy cells with high mitochondrial of ΔΨm, the dye reagent aggregates and emits red fluorescence (normal membrane potential). When the ΔΨm collapses, JC-1 can no longer accumulate within the mitochondria, which shows only green (loss membrane potential).

### 3.9. RNA Extraction and Reverse Transcription-Polymerase Chain Reaction (RT-PCR)

Total RNA was extracted from Hela and SW1353 cells for treated with and without gliotoxin. Cells in 6 cm^2^ dishes were lysed with 200 µL of TRIzol^®^ reagent following the manufacturer’s recommendations. RT-PCR was performed to check specific mRNA expression in differentiated cells. One µg of total RNA was mixed diethylpyrocarbonate (DEPC)-treated water to reach the total volume of 19 µL in Maxime PreMix Kit, was reverse transcription reaction 45 °C for 30 min and inactivation of RTase 94 °C for 5 min. The target cDNA was amplified using the following primers: forward 5′-CCC-AGG-CCG-TGA-GGA-GTT-AGC-3′ and reverse 5′-CAG-CAT-CAC-TGT-AAC-TTG-CTA-ATC-3′ for caspase-3; forward 5′-CAC-TAG-AAA-GGA-GGA-GAT-GGA-AAG-3′ and reverse 5′-CTA-TCC-TGT-TCT-CTT-GGA-GAG-TCC-3′ for caspase-8; forward 5′-GCT-CTT-CCT-TTG-TTC-ATC-TCC-3′ and reverse 5′-CAT-CTG-GCT-CGG-GGT-TAC-TGC-3′ for caspase-9; forward 5′-GGA-GGC-AAG-CAT-AAG-ACT-GG-3′ and reverse 5′-GTC-TGC-CCT-TTC-TCC-CTT-CT-3′ for cyt c; forward 5′-TGC-CAG-CAA-ACT-GGT-GCT-CA-3′ and reverse 5′-GCA-CTC-CCG-CCA-CAA-AGA-TG-3′ for Bax; forward 5′-CGC-ATC-AGG-AAG-GCT-AGA-GT-3′ and reverse 5′-AGC-TTC-CAG-ACA-TTC-GGA-GA-3′ for Bcl-2; forward 5′-GCG-CAC-AGA-GGA-AGA-GAA-TC-3′ and reverse 5′-CTC-TCG-GAA-CAT-CTC-GAA-GC-3′ for p53; forward 5′-GAA-GGT-CGG-AGT-CAA-CGG-ATT-T-3′ and reverse 5′-ATG-GGT-GGA-ATC-ATA-TTG-GAA-C-3′ for GAPDH [33,34]. PCR products were electrophoresed on 1.0% agarose gel and visualized by ethidium bromide staining under ultra-violet light.

### 3.10. Protein Extraction and Western Blotting Assay

Hela and SW1353 cells were treated with different concentrations of gliotoxin. The cells were harvested and then lysed with RIPA buffer [50 mM Tris-HCl (pH 7.5), 150 mM NaCl, 1% Triton X-100, 1% sodium deoxycholate, 2 mM EDTA (pH 8.0), and 0.1% SDS]. For separate extraction of cytoplasmic proteins, Cytosol Fractionation Kit of BioVision (Milpitas, CA, USA) was used following manufacturer’s instructions. After 13,000 × *g* centrifugation at 4 °C for 15 min, protein concentration was determined by the bicinchoninic acid (BCA) method using bovine serum albumin as standard. Forty micrograms of total cellular proteins were separated by sodium dodecyl sulfate polyacrylamide gel electrophoresis (SDS-PAGE) (12% for caspase-3, caspase-8, caspase-9 and p53; 15% for Bcl-2, Bax, and cyt c), transferred onto a polyvinylidene difluoride (PVDF) membranes (Millipore, Billerica, MA, USA). Membranes were blocked with 5% skim milk in Tris buffered saline containing Tween-20 (TBS-T) (20 mM Tris-HCl, pH 7.6, 136 mM NaCl, and 0.1% Tween-20) for 2 h and then incubated with primary antibody (1:500 dilution) in blocking agent at 4 °C overnight. After washing with TBS-T buffer, the membrane was incubated with secondary antibody (1:5000 dilution) for 1 h at room temperature. Bands were visualized by enhanced chemiluminescence and LAS-4000 imaging system (FUJIFILM, Tokyo, Japan).

### 3.11. Statistical Analysis

The results are presented as the mean ± standard deviation. Significant differences among the groups were determined using the unpaired Student’s *t*-test. The differences were considered statistically significant at *p* < 0.05.

## 4. Conclusions

In conclusion, the data reveal that HeLa and SW1353 cells are highly sensitive to growth inhibition and apoptosis induction by gliotoxin. These results indicated that gliotoxin induces apoptosis through the recruitment of caspase-8 will be activated and it is able to directly activate caspase-3, an effector protein. The mitochondrial pathways mediated by down-regulation of Bcl-2 and up-regulation of Bax the release of cyt c. Following its formation, the complex will activate caspase-9, an initiator protein. In return, the activated caspase-9 works together with the complex of cyt c, which in turn activates caspase-3 to apoptotic mode of cell death. Briefly, the present study examined that gliotoxin induced apoptotic cell death in association with activating expressions of caspase family enzymes followed by cyt c, Bax and Bcl-2 regulation. Further, studies will be required to investigate the effect of gliotoxin on mitochondria during apoptosis in HeLa and SW1353 cells.
